# Production of eurycomanone from cell suspension culture of *Eurycoma longifolia*

**DOI:** 10.1080/13880209.2017.1400077

**Published:** 2017-11-12

**Authors:** Nguyen Huu Nhan, Nguyen Hoang Loc

**Affiliations:** a College of Sciences, Institute of Bioactive Compounds, Hue University, Hue, Vietnam;; b College of Food Industry, Da Nang, Vietnam

**Keywords:** Callus, quassinoid, Simaroubaceae

## Abstract

**Context:** Eurycomanone is found in the *Eurycoma longifolia* Jack (Simaroubaceae) tree, exhibits significant antimalarial activity, improves spermatogenesis, suppresses expression of lung cancer cell tumour markers and regulates signalling pathways involved in proliferation, cell death and inflammation.

**Objectives:** Establishment of cell suspension culture of *E. longifolia* to determine the eurycomanone accumulation during cultures.

**Materials and methods:** Callus of *E. longifolia* was cultured in MS medium supplemented with 0.8% agar, 30/L sucrose, 1.25 mg/L NAA and 1 mg/L KIN for biomass production. Cell suspension culture was established by transferring friable calli to the same medium without agar. Eurycomanone content during cell culture was determined by HPLC with a C18 column, flow rate of 0.8 mL/min, run time of 17.5 min, detector wavelength of 254 nm. The stationary phase was silica gel and the mobile phase was acetonitric:H_2_O. Roots of 5 year-old trees were used as the control.

**Results:** The cells from 3 g of inoculum increased in biomass with a maximum value of 16 g fresh weight (0.7 g dry weight) at 14th day of culture. The cell growth then decreased from day 14 to day 20. Eurycomanone was produced during culture from the beginning to 20th day, its highest content (1.7 mg/g dry weight) also obtained at 14th day (the control is 2.1 mg/g dry weight).

**Discussion and conclusions**: Cell suspension culture of *E. longifolia* is a suitable procedure to produce eurycomanone. The yield of eurycomanone biosynthesis in 14 days-old cells are relatively high, approximately 0.8 times the control.

## Introduction

*Eurycoma longifolia* Jack (Simaroubaceae), one of the most popular tropical medicinal plants, is found in Indonesia, Malaysia, Vietnam, Cambodia, Myanmar and Thailand.

According to Bhat and Karim (2010), the root of *E. longifolia* is the most valuable component and its extract is used in traditional medicine of Southeast Asian region for the treatment diseases such as persistent fever, malaria, aches, dysentery, sexual insufficiency, restoring energy and vitality, enhancing blood flow, and as a herbal ingredient for women after child birth.

The extract of *E. longifolia* plant parts (particularly roots) displayed antimalarial, antimicrobial, antipyretic, antiulcer, antidiabetic, aphrodisiac activities and cytotoxicity against cancerous cells (Tada et al. 1991; Chan et al. 2004; Kuo et al. 2004; Bhat and Karim 2010). Besides, several clinical studies have shown that the sperm quality of idiopathic infertile rats and low level of testosterone were improved when treated with the plant aqueous extract (Tambi and Imran 2010; Tambi et al. 2012).

Many reports show that *E. longifolia* parts are rich in bioactive compounds, mainly alkaloids such as 9-methoxycanthin-6-one, 9-methoxycanthin-6-one-*N*-oxide, 9-hydroxycanthin-6-one and 9-hydroxycanthin-6-one-*N*-oxide (Kardono et al. 1991), and quassinoids such as eurycomaoside, eurycolactone, eurycomalactone, eurycomanone and pasakbumin-B (Bhat and Karim 2010).

Eurycomanone, a bioactive quassinoid, exhibits significant antimalarial activity against *Plasmodium falciparumi* William H. Welch (Plasmodiidae) strains (Chan et al. 1986, 2004; Kardono et al. 1991), improves spermatogenesis of rat by enhancing production of testosterone (Low et al. 2013). Besides, eurycomanone also can suppress expression of lung cancer cell tumour markers (Wong et al. 2012) and regulate signalling pathways involved in proliferation, cell death and inflammation (Hajjouli et al. 2014).

There were some reports on accumulation of bioactive metabolites in *in vitro* cultures of *E. longifolia* such as 9-methoxycanthin-6-one from hairy roots (Abdullah et al. 2016) or from calli (Mahmood et al. 2011); canthinone (Siregar et al. 2009), alkaloids (Keng et al. 2010; Galih and Esyanti 2014) or some other compounds (Natanael et al. 2014) from cells. Iriawatia et al. (2014) also found 3-[(cyclohexyl-methyl-amino)-methyl]-3H-benzooxazole-2-one and 2-furancarboxaldehyde, 5-(hydroxymethyl) in embryogenic calli and somatic embryos. Lulu et al. (2015) produced 2,2-diphenyl-1-picrylhydrazyl and flavonoid from adventitious root cultures.

However, up to now we have not found any reports on biosynthesis of eurycomanone in cell suspension cultures of *E. longifolia*. This present work, therefore, investigated suitable medium for cells biomass production and their eurycomanone accumulation.

## Materials and methods

### Callus culture

Callus of *E. longifolia* that was provided by Dr. Tuan VC (College of Education, Danang University, Vietnam) was transferred on MS (Murashige and Skoog 1962) medium supplemented with 0.8% agar, carbon sources (fructose, glucose and sucrose) and plant growth regulators (naphthaleneacetic acid – NAA, and kinetin – KIN) at various concentrations to find suitable medium for biomass production.

Two-week old calli were subcultured and maintained on the most suitable medium. The medium was adjusted to pH 5.8. The cultures were incubated at 25 ± 2 °C under intensity of 2000–3000 lux with a photoperiod of 8 h day light.

### Cell suspension culture

Cell suspension culture was established by agitation of callus in a 250 mL Erlenmeyer flask containing 50 mL of callus culture medium at a shaking speed of 120 rpm under the same conditions as for the callus culture except the intensity of 500 lux.

Samples were obtained every two days until 20 days to determine fresh and dry weight of the cell biomass. To measure fresh weight, suspension cells were filtered, washed with distilled water, collected, and weighed. The dry weight was determined by drying the fresh cells at 50 °C until a constant weight was attained.

Growth index (GI) was calculated as:

GI=Final fresh cell weight/Initial fresh cell weight

### Eurycomanone quantification

The dried cell biomass was powdered to fine particles, 0.5 g of this powder was soaked in 10 mL methanol at 60 °C and with a shaking speed of 120 rpm for 8 h, repeating this step for three times. The extract (30 mL) was then filtered and concentrated completely at 50 °C. The precipitate was dissolved in 5 mL methanol (eurycomanone extract) and filtered through Minisart 0.2 µm membrane (Sartorius, Goettingen, Germany) to prepare sample for HPLC.

The HPLC analysis was carried out at ambient temperature with a C18 column (Xbridge: 5 µm, 4.6 × 250 mm), flow rate: 0.8 mL/min, run time: 17.5 min, detector wavelength: 254 nm. The stationary phase was silica gel and the mobile phase was acetonitric:H_2_O (15:85). A 20 µL aliquot of sample was injected into the column using a Hamilton syringe. The HPLC was performed on a LC-20 Prominence system (Shimadzu, Kyoto, Japan) with a SPD-20A UV-VIS detector using LC-Solution software. All solvents were of analytical grade and were purchased from Merck & Co. Inc. (Darmstadt, Germany).

A standard curve of eurycomanone (Santa Cruz, CA) was used for measurement of the eurycomanone content in the samples.

### Statistical analysis

All experiments were repeated at least three times. The data are represented as the mean of the repeats; the means were compared using a one-way analysis of variance followed by Duncan’s test (*p* < 0.05).

## Results and discussion

### Callus culture

Effect of plant growth regulators as NAA and KIN on callus growth of *E. longifolia* was observed in the present work. The response of explants cultured on MS media with 30 g/L sucrose containing different concentrations of NAA is shown in Table 1. NAA at 1.25 mg/L level resulted in highest efficiency, callus biomass from 3 g of inoculum/flask reached 23.59 g of fresh weight (0.93 g of dry weight) with *p* < 0.05 and GI of about 7.8. The lowest callus biomass was recorded in medium with NAA from 2 to 2.25 mg/L, 16.79 to 17.24 g of fresh weight (*p* > 0.05) corresponding to 0.62–0.65 g dry weight (*p* < 0.05).

**Table 1.** Effect of NAA on callus growth after 2 weeks of culture.

NAA (mg/L)	Fresh weight (g)	Dry weight (g)	GI	Callus morphogenesis
0.50	20.47^c^	0.79^d^	6.8	Light yellow and soft
0.75	21.92^e^	0.88^e^	7.3	Light yellow and soft
1.00	21.30^d^	0.87^e^	7.1	Yellow and friable
1.25	23.59^f^	0.93^f^	7.8	Yellow and friable
1.50	18.50^b^	0.72^c^	6.2	Yellow and friable
1.75	18.21^b^	0.71^c^	6.1	Yellow and friable
2.00	17.24^a^	0.65^b^	5.7	Light yellow and soft
2.25	16.79^a^	0.62^a^	5.6	Light yellow and soft

Different letters in a column indicate significantly different means (Duncan’s test, *p* < 0.05).

**Figure 1.**
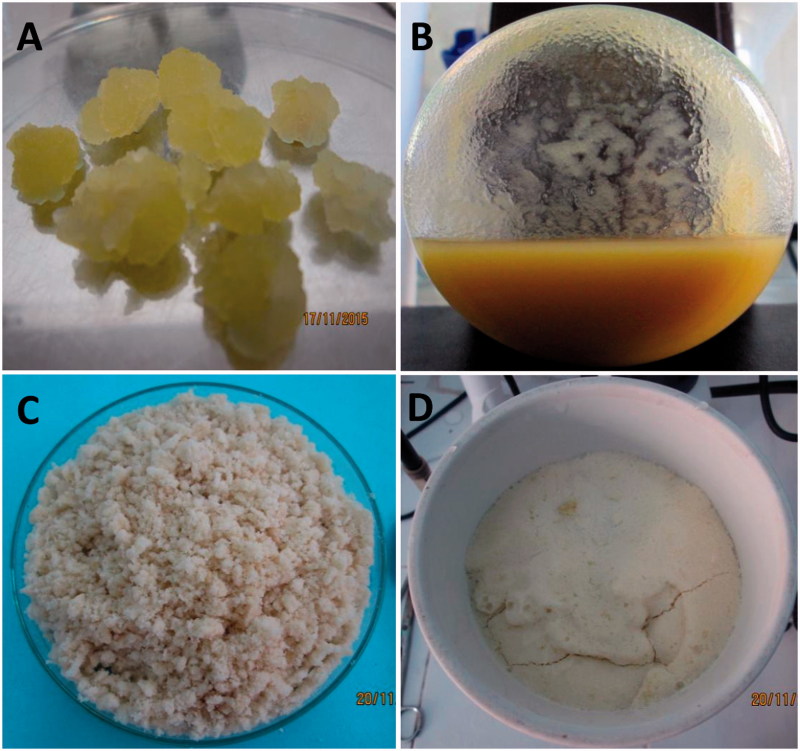
*In vitro* cultures of *E. longifolia*. (A) Callus, (B) suspension cell, (C) fresh cell biomass of 14 days-old culture after filtration and (D) fresh cell biomass after vacuum filtration.

Among the various combinations of 1.25 mg/L NAA and KIN, highest callus biomass of (30.98–32.34 g of fresh weight/1.26–1.31 g of dry weight, *p* > 0.05) was obtained on MS medium containing 0.75–1 mg/L KIN with GI reached 10.3–10.7 (Table 2, Figure 1(A)). This result indicates that the addition of NAA in combination with KIN further promotes the callus growth compares to NAA applied singly.

**Table 2.** Effect of NAA (1.25 mg/L) and KIN on callus growth after 2 weeks of culture.

KIN (mg/L)	Fresh weight (g)	Dry weight (g)	GI	Callus morphogenesis
0.50	23.43^b^	0.92^b^	7.8	Light yellow and soft
0.75	30.98^cd^	1.26^d^	10.3	Light yellow and soft
1.00	32.34^d^	1.31^d^	10.7	Yellow and friable
1.25	28.80^c^	1.11^c^	9.6	Yellow and friable
1.50	24.65^b^	0.94^b^	8.2	Yellow and friable
1.75	20.49^a^	0.79^a^	6.8	Light yellow and soft
2.00	18.70^a^	0.78^a^	6.2	Light yellow and soft

Different letters in a column indicate significantly different means (Duncan’s test, *p* < 0.05).

The reports showed that a suitable ratio of auxin and cytokinin in medium promoted strongly callus proliferation in various plant species. For example, O’Dowd et al. (1993) produced callus of nine various species belonging to *Ephedra* genus on modified MS medium supplemented with 0.25 μM KIN and 5.0 μM 2,4-D (2,4-dichlorophenoxyacetic acid) or NAA. Both media were then used to establish cell suspension cultures for several species of this genus. Balbuena et al. (2009) also produced callus biomass of *Piper solmsianum* C. DC. (Piperaceae) on MS medium with 0.2 mg/L 2,4-d and 2 mg/L BA. Zakaria et al. (2011) induced and produced callus biomass of *Papaver orientale* L. (Papaveraceae) on the same MS medium containing 0.5 mg/L BA (benzyladenine) and 0.5 or 1 mg/L NAA. According to Adhikari and Pant (2013), the best growth of *Withania somnifera* (L.) Dunal (Solanaceae) callus was observed on the MS medium supplemented with 0.5 mg/L BAP and 1.5 mg/L NAA after eight weeks of culture.

For *E. longifolia*, Siregar et al. (2003) induced callus from leaves of nine different trees on MS medium with 10 mg/L NAA. After four weeks of culture, callus reached to 2.723–3.816 g fresh weight (insignificant difference) with respective dry weight of 0.106–0.141 g. Callus was maintained in the same medium by subculturing every four weeks.

### Cell growth and eurycomanone accumulation

Cell suspension culture was carried out in liquid MS medium supplemented with 30 g/L sucrose, 1.25 mg/L NAA and 1 mg/L KIN (Figure 1(B)).

**Figure 2.**
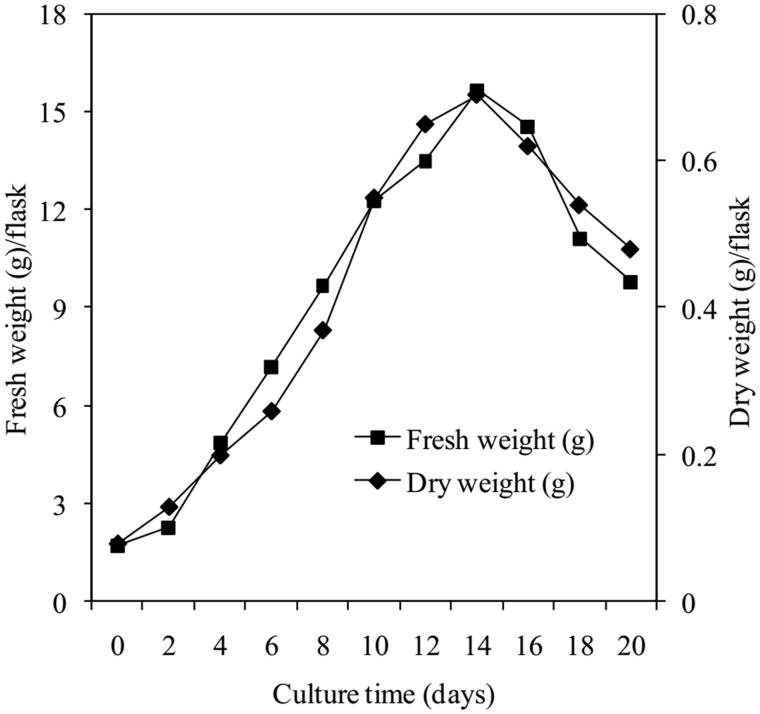
Time course of *E. longifolia* cell growth.

Results in Figure 2 show that the growth curve of *E. longifolia* cells forming a sigmoid pattern. During 0–2th days, the biomass of cell-aggregate (approximately 1.7 g of inoculum) increased slowly, then they entered the logarithmic phase on 2th to 14th day as indicated by a rapid increase in biomass with a maximum value of approximately 16 g fresh weight (0.7 g dry weight, *p* < 0.05) (Figure 1(C,D)), the cell growth decreased from 14th to 20th day.

Siregar et al. (2004) cultured the cell suspension of *E. longifolia* on MS medium with 1 mg/L NAA. The results showed from 1 g of inoculum they obtained approximately 5 g fresh weight (0.3 g dry weight). Keng et al. (2010) obtained highest biomass (3.1 g fresh weight/0.22 g dry weight) at 13th day when cultured 0.5 g cells of *E. longifolia* in MSB medium (a modified MS medium) treated with 2 mg/L NaH_2_PO_4_. Natanael et al. (2014) multiplied callus in solid MS medium with 2.2 ppm 2,4-d and 2 ppm KIN. Cell culture was established by transferring the callus from the solid culture into a liquid medium with 1.1 ppm 2,4-d and 1 ppm KIN. *E. longifolia* callus was also maintained in solid Zenk medium added with 0.5 ppm NAA and 0.5 ppm KIN. Calli were then subcultured three times into the same medium without agar to obtain cell biomass. Maximum cell biomass reached nearly 0.25 g after 16 days of culture (Galih and Esyanti 2014).

**Figure 3.**
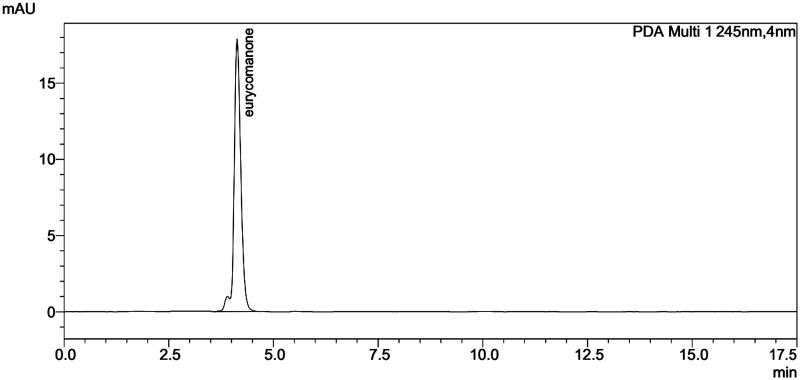
HPLC chromatogram of standard eurycomanone.

**Figure 4.**
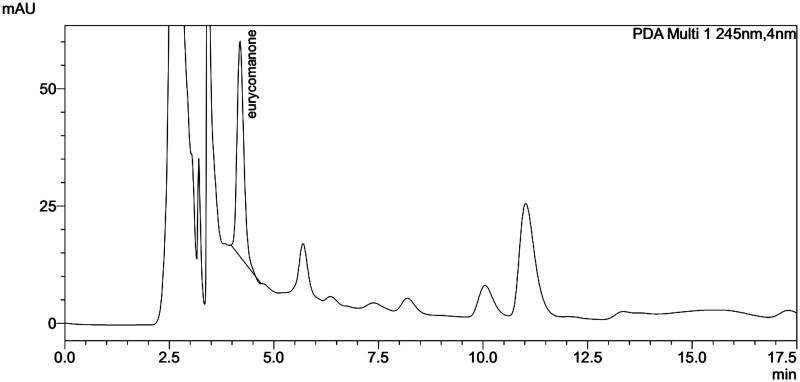
HPLC chromatogram of eurycomanone extract from 14 days-old cell aggregate of *E. longifolia*.

**Figure 5.**
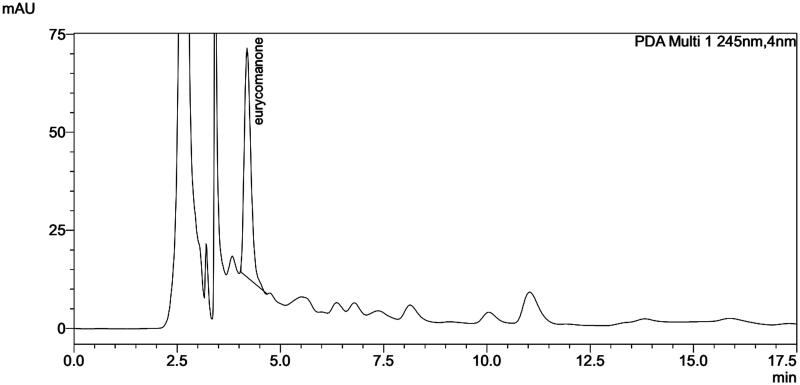
HPLC chromatogram of eurycomanone extract from *E. longifolia* rhizome.

HPLC analysis was performed on the extract of cell-aggregate and the results showed a peak with a retention time of 4.194 min similar to that of the standard eurycomanone (4.141 min) and the rhizome (4.198 min). Generally speaking, the HPLC chromatograms of the extract of cell and rhizome are in the same pattern (Figures 3–5). The eurycomanone found in *in vitro* cultured cells of *E. longifoli*a and the biotransformation seems insignificant.

**Figure 6.**
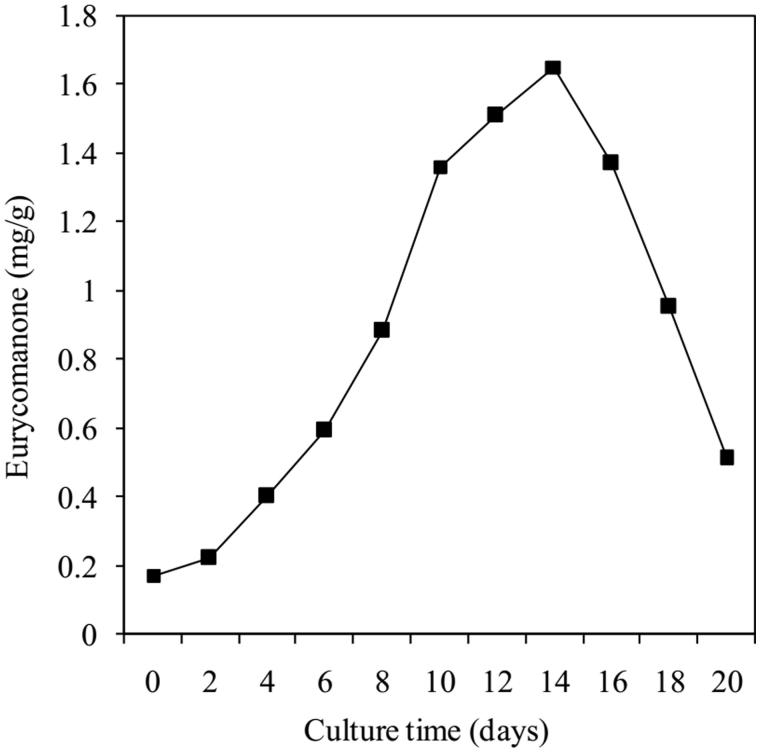
Time course of eurycomanone accumulation in *E. longifolia* cells.

Figure 6 shows that eurycomanone compound was produced during culture from the beginning to 20th day, its highest content (approximately 1.7 mg/g dry weight, *p* < 0.05) also obtained at 14th day like the accumulation of cell biomass. The control (roots of 5 years-old tree) is approximately 2.1 mg/g. According to Mohamada et al. (2013), eurycomanone content of 6–7 years-old *E. longifolia* trees collected from Muar (Johor, Malaysia) is only nearly 1.4 mg/g.

### Effect of carbon sources on cell growth and eurycomanone accumulation

Carbohydrates are known to influence the cell growth and the production of phytochemicals in *in vitro* cultures. The study of Hippolyte et al. (1992) showed that the maximum yield of rosmarinic acid produced by cell suspension cultures of *Salvia officinalis*L. (Lamiaceae) was 3.5 g/L when 5% of sucrose was used but it was only 0.7 g/L in the medium containing 3% sucrose. Liang et al. (2004) investigated effects of sucrose concentrations (2–5%) on the production of puerarin and isoflavones compounds in hairy-root culture of *Pueraria phaseoloides* (Roxb.) *Benth.* (Fabaceae). The results showed that 3% sucrose in medium was the best for accumulation of puerarin and isoflavones. The highest content of puerarin (5.147 mg/g dry weight) was obtained after 12 days of culture while the highest content of isoflavones (about 27.76 mg/g dry weight) was gained after 16 days. According to Qian et al. (2009), the rosmarinic acid accumulation was enhanced by the sucrose concentrations added in the culture medium of *Coleus blumei* Benth. (Lamiaceae). When the sucrose content was 6%, the calli and cells accumulated 33.7% and 10.1% (dry weight) rosmarinic acid, respectively. Higher sucrose content (≥5%) had a negative influence on their growth. Manuhara et al. (2015) indicated that various concentrations (3–6%) of sucrose influenced the biomass and saponin content in hairy-root culture of *Talinum paniculatum* (Jacq.) Gaertn. (Talinaceae). Maximum biomass was reached on MS medium supplemented with 6% sucrose is approximately three-fold higher than control. However, maximum saponin content was obtained on MS medium supplemented with 5% sucrose. Siregar et al. (2009) investigated effect of sucrose concentration (0–7%) on the growth of *E. longifolia* cells in modified liquid MS medium. The results showed that 4% sucrose increased fresh biomass up to 7 g/25 mL flask (approximately 0.6 g dry biomass).

The present work also resulted in similarity to the above. Data in Table 3 show that sucrose concentrations (from 20 to 40 g/L) in the medium did not affect fresh cell biomass (17.43–18 g, *p* > 0.05), but the biomass of dry cell changed significantly (*p* < 0.05). The concentration of 30 g/L sucrose as used in the previous experiments is still the most suitable, the maximum dry biomass reached 0.72 g. The eurycomanone production of the cells is also promoted strongly (1.7 mg/g) at this sucrose concentration. The effect of glucose concentration on the growth and eurycomanone accumulation was found to be lower than that of sucrose. Meanwhile, the cells did not grow in the media using fructose as a carbon source (data not shown).

**Table 3.** Effect of carbon sources on cell growth and eurycomanone accumulation after 14 days of culture.

	Glucose (g/L)			Sucrose (g/L)		
Characteristics	20	30	40	20	30	40
Fresh weight (g)	17.58^a^	20.30^b^	12.57^c^	17.62^b^	17.43^b^	18.00^b^
Dry weight (g)	0.43^a^	0.59^b^	0.44^a^	0.44^a^	0.72^c^	0.61^b^
Eurycomanone (mg/g)	0.47^a^	0.77^b^	1.36^d^	1.54d^e^	1.70^e^	1.11^c^

Different letters in a column indicate significantly different means (Duncan’s test, *p* < 0.05).

## Conclusions

In our research, 3 g of cell biomass cultured in liquid MS medium supplemented with 30 g/L sucrose, 1.25 mg/L NAA and 1 mg/L KIN increased in maximum biomass up to 16 g fresh weight (0.7 g dry weight) at 14th day. Eurycomanone was produced during culture from the beginning to 20th day, its highest content (1.7 mg/g dry weight) also obtained at 14th day. Cell suspension culture of *E. longifolia* is a suitable procedure to produce eurycomanone. The yield of eurycomanone biosynthesis in 14 days-old cells is relatively high, approximately 0.8 time in comparison with roots of 5 years-old tree.
